# Independent Component Analysis and Graph Theoretical Analysis in Patients with Narcolepsy

**DOI:** 10.1007/s12264-018-0307-6

**Published:** 2018-11-13

**Authors:** Fulong Xiao, Chao Lu, Dianjiang Zhao, Qihong Zou, Liyue Xu, Jing Li, Jun Zhang, Fang Han

**Affiliations:** 1 Sleep Medicine Center, Department of Respiratory and Critical Care Medicine, Peking University People’s Hospital, Beijing, 100044 China; 2 Department of Radiology, Peking University International Hospital, Beijing, 102206 China; 3 Center for MRI Research, Academy for Advanced Interdisciplinary Studies, Peking University, Beijing, 100871 China; 4 PKU-UPenn Sleep Center, Peking University International Hospital, Beijing, 102206 China; 5 Department of Neurology, Peking University People’s Hospital, Beijing, 100044 China

**Keywords:** Narcolepsy, Functional connectivity, Independent component analysis, Graph theory

## Abstract

The present study was aimed to evaluate resting-state functional connectivity and topological properties of brain networks in narcolepsy patients compared with healthy controls. Resting-state fMRI was performed in 26 adult narcolepsy patients and 30 matched healthy controls. MRI data were first analyzed by group independent component analysis, then a graph theoretical method was applied to evaluate the topological properties in the whole brain. Small-world network parameters and nodal topological properties were measured. Altered topological properties in brain areas between groups were selected as region-of-interest seeds, then the functional connectivity among these seeds was compared between groups. Partial correlation analysis was performed to evaluate the relationship between the severity of sleepiness and functional connectivity or topological properties in the narcolepsy patients. Twenty-one independent components out of 48 were obtained. Compared with healthy controls, the narcolepsy patients exhibited significantly decreased functional connectivity within the executive and salience networks, along with increased functional connectivity in the bilateral frontal lobes within the executive network. There were no differences in small-world network properties between patients and controls. The altered brain areas in nodal topological properties between groups were mainly in the inferior frontal cortex, basal ganglia, anterior cingulate, sensory cortex, supplementary motor cortex, and visual cortex. In the partial correlation analysis, nodal topological properties in the putamen, anterior cingulate, and sensory cortex as well as functional connectivity between these regions were correlated with the severity of sleepiness (sleep latency, REM sleep latency, and Epworth sleepiness score) among narcolepsy patients. Altered connectivity within the executive and salience networks was found in narcolepsy patients. Functional connection changes between the left frontal cortex and left caudate nucleus may be one of the parameters describing the severity of narcolepsy. Changes in the nodal topological properties in the left putamen and left posterior cingulate, changes in functional connectivity between the left supplementary motor area and right occipital as well as in functional connectivity between the left anterior cingulate gyrus and bilateral postcentral gyrus can be considered as a specific indicator for evaluating the severity of narcolepsy.

**Electronic supplementary material:**

The online version of this article (10.1007/s12264-018-0307-6) contains supplementary material, which is available to authorized users.

## Introduction

Narcolepsy is a chronic sleep disorder with the main symptoms of excessive daytime sleepiness (EDS), accompanied by the occurrence of rapid eye-movements (REM) and daytime sleep attacks, especially in monotonous situations [1]. Other clinical manifestations of narcolepsy include cataplexy, sleep paralysis, and hypnagogic or hypnopompic hallucinations. The pathology of narcolepsy is caused by the apoptosis of hypothalamic neurons producing hypocretin, a wake-promoting neurotransmitter that can be measured in the cerebrospinal fluid [2, 3]. Sleep-onset REM is a characteristic of the sleep architecture in narcolepsy patients due to the dysfunctional hypocretin [1]. It has been demonstrated that narcolepsy patients have an 85%–95% reduction in the number of hypocretin neurons [4, 5]. These neurons project extensively to most areas of the brain, and it has been demonstrated that hypocretin and its receptors play important roles in many physiological activities, such as feeding, energy homeostasis, the sleep-wake cycle, and neuroendocrine systems [2].

Qualitative MRI examination of the brain is usually negative in primary narcolepsy, so functional MRI has been used to detect the underlying neuronal abnormality. Alterations in cortical thickness, resting/task state, and metabolism have been reported in narcolepsy patients by neuroimaging research [6–10], but the results are controversial in some conditions. The resting-state network (RSN), which is based on functional connectivity (FC) in the resting state, exhibits blood oxygen level-dependent (BOLD) signal fluctuations in brain areas [11, 12]. The RSN can be revealed by a data-driven method called independent component analysis (ICA), in which some separate brain regions are connected to each other to form a network by calculating the correlations of time series from the BOLD signal [13, 14]. ICA is a statistical method to discover the hidden spatiotemporal components contained within brain imaging data [15]. Researchers do not need a hypothesis or to select a reference region using ICA. All components of activation from ICA results are statistically independent of each other.

In addition to ICA, graph theoretical analysis can also describe the properties of a brain network, considering the whole connectivity among all areas [16, 17]. Graph theory is a widely-applicable method of measuring relationships in data [18]. It has been frequently used to assess both the functional and structural connectivity in brain imaging data. In graph-based analysis, the brain is regarded as an entire network composed of nodes and edges. A node in the network represents a specific region, and an edge represents the FC between each pair of regions. Then the relationships between each pair of regions (nodes) can be stored in an *n* × *n* matrix, each row or column corresponding to one of the areas in the parcellation [18]. Once the matrix has been constructed, the properties, such as path length, modularity, efficiency and others can be calculated to describe the global or local network topology [19]. Graph-based network analysis is important for describing the topological properties of the network, in which the neural activity in the brain can be interpreted from the network and information-processing viewpoint. Also in the analysis of topological properties, the functional connections between two regions by analyzing time series correlations in regional activity can shed light on potential physiological or disease processes. To study differences in FC between groups, either individual connections between brain regions or global network connections have been analyzed.

Previous studies of narcolepsy with cataplexy using BOLD MRI have mainly focused on the abnormal activation during emotional processing. Areas associated with emotional regulation, such as the amygdala, anterior cingulate, and prefrontal-orbitofrontal cortices, exhibit significant differences between narcolepsy patients and healthy controls [20, 21]. But the results of these studies remain controversial [20, 21]. Furthermore, BOLD MRI in narcolepsy with cataplexy during resting wakefulness has not been well explored. Based on the previous studies, our hypothesis was that the hypocretin deficiency in narcolepsy may be reflected by abnormal connectivity within the whole brain during resting wakefulness and the abnormality may be associated with the severity of sleepiness in narcolepsy with cataplexy.

This study was designed to illuminate changes in the resting state FC and topological properties in adult narcolepsy patients, as well as the relationship between clinical variables (severity of sleepiness) and functional plasticity in narcolepsy.

## Materials and Methods

### Participants

We recruited 26 adult patients, newly diagnosed as suffering from narcolepsy with cataplexy according to the International Classification of Sleep Disorders (ICSD)-3 [22], from the Sleep Medicine Center of the Respiratory Department at Peking University People’s Hospital between November 2016 and February 2018. Another 30 gender- and age-matched healthy adults were recruited from the community (Table 1). None of the healthy adults suffered from psychiatric or neurological disorders. All adult narcolepsy cases were first-time visitors and had never taken psychiatric stimulants. According to the International Classification of Sleep Disorders criteria for narcolepsy, the clinical diagnosis was made by a sleep specialist based on both EDS lasting > 3 months and a defined history of cataplexy. The final diagnosis was confirmed by a nocturnal polysomnogram followed by the multiple sleep latency test (MSLT). The Epworth Sleepiness Score (ESS) was used to measure the severity of EDS in both narcolepsy patients and healthy controls. In the ESS, the participant was required to describe the possibility of falling asleep on a scale from 0 to 3 for eight different situations; a total score ≥ 10 suggests EDS [23].

**Table 1:** Demography of narcolepsy patients and healthy controls.

	Narcolepsy patients	Healthy controls	*P* value
Age	25.77 ± 6.64	25.37 ± 4.31	0.786
Gender (male/female)	18/8	18/12	0.58
BMI	25.93 ± 4.10	26.14 ± 2.71	0.82
ESS	17.54 ± 4.39	5.4 ± 1.52	< 0.001
Mean sleep latency (min)	0.68 (0.28, 1.28)	–	–
Mean REM sleep latency (min)	0.75 (0, 2.58)	–	–

The *P* value for gender distribution in the two groups was obtained using the *χ*^2^ test, and for differences in age and BMI in the two groups by the two-sample *t* test. Values are expressed as the mean ± SD or median (25% quartile, 75% quartile). BMI, body mass index; ESS, Epworth Sleepiness Score. Average sleep latency and REM sleep latency were calculated from the five naps in the MSLT.

The exclusion criteria for both narcolepsy patients and healthy adults were as follows: (1) other sleep disorders, such as obstructive sleep apnea or insomnia; (2) diabetes, chronic pulmonary/respiratory disease, or heart disease; (3) neurological diseases and structural brain lesions based on imaging; (4) psychiatric disorders; (5) alcohol, drug, or substance abuse; (6) congenital or inherited diseases; or (7) MRI contraindications, such as claustrophobia or metal implants.

This research was performed in accordance with the ethical guidelines of the Declaration of Helsinki (version 2002) and was approved by the Medical Ethics Committee of Peking University People’s University. All participants provided written informed consent.

### Polysomnography and Excessive Daytime Sleepiness Evaluation

All patients were required to abstain from coffee or alcoholic drinks for one day prior to participation in this study. Full nocturnal polysomnography was recorded on a Respironics LE-Series Physiological Monitoring System (Alice 6 LE, Philips, Murrysville, FL) one day before MRI examination. Polysomnography was recorded from 22:00 until 06:00. Based on the American Academy of Sleep Medicine guidelines, we recorded the standard electroencephalogram (EEG from frontal, central, and occipital regions: F4/M1, C4/M1, and O2/M1, and back-up F3/M2, C3/M2, and O1/M2), chin electromyograms (EMG from 3 chin electrodes and the middle of the right anterior tibialis), the electro-oculogram (EOG located in the cornea and retina), and electrocardiogram. The oral and nasal airflow, snoring, chest and abdominal breathing, oxygen saturation, and body position were also recorded, along with total sleep time, sleep latency, sleep efficiency, arousal, and respiratory events. According to the American Academy of Sleep Medicine manual, obstructive apnea was defined as a reduction in airflow ≥ 90% lasting at least 10 s and associated with persistent respiratory effort; hypopnea was defined as a reduction in airflow ≥ 30% lasting at least 10 s and accompanied by a 4% or greater oxygen desaturation [24]. The Apnea–Hypopnea Index was calculated as the average of the total number of apnea and hypopnea events experienced per hour of sleep. All patients were asked to complete a clinical MSLT on the day after the nocturnal polysomnography. As in the American Academy of Sleep Medicine task-force-approved modification [25], naps were scheduled at 2-h intervals starting 2 h after initial morning awakening. If no sleep occurred in 20 min, the nap trial ended and sleep latency was recorded as 20 min. If sleep occurred within 20 min, onset was defined as the time from lights-out to the first epoch of sleep (including stage 1). In order to assess the presence of REM sleep, the test continued for ≥ 15 min after sleep onset. If present, the latency to REM sleep was also recorded. Then the average sleep latency and REM sleep latency from the 5 naps in MSLT were calculated.

### Imaging Data Acquisition

MRI examination was performed immediately following the clinical MSLT. MRI data were obtained on a 3T scanner (Siemens, Skyra, Germany) using an 8-channel brain phased-array coil. Foam pads were used to minimize head motion, and headphones were used to reduce scanner noise. Resting BOLD MRI scans were obtained with gradient-echo planar imaging (TR = 2030 ms, TE = 30 ms, slice = 33, FA = 90°, FOV = 224 mm × 224 mm, matrix = 64 × 64, voxel size = 3.5 × 3.5 × 3.5). After the BOLD MRI scan, a high-resolution T1-weighted structural image was acquired with the following parameters: TR = 1900 ms, TE = 2.55 ms, FA = 9°, FOV = 240 mm × 240 mm, thickness = 1 mm. All patients and controls were asked to resist sleeping in order to remain fully awake [6, 26], not to move, and to keep the eyes open during the whole MRI scan, supervised clinically and by video during the whole process. In addition, emotional triggering factors were avoided during the whole process to prevent a cataplexy attack.

### Preprocessing of Functional Imaging Data

Functional MRI data preprocessing was performed using the Data Processing and Analysis for Resting State Brain Imaging V2.1 (DPABI V2.1 [27]), which works with Statistical Parametric Mapping (SPM8) implemented in the MatLab platform (The MathWorks Inc., Natick, MA). A total of 240 functional volumes were acquired in the resting BOLD MRI scans. The first 5 functional volume images of each participant’s dataset were discarded, then the remaining fMRI data were corrected for slice timing and realigned for motion correction. Participants with head motion exceeding 3 mm in translation and 3° in rotation were rejected. Anatomical and functional images were first manually reoriented to the anterior commissure, and structural images were co-registered to the functional images for each participant using a linear transformation. Then the co-registered functional images were normalized to the standard Montreal Neurological Institute space template with a resampling voxel size of 3 mm × 3 mm × 3 mm. The normalized functional images were smoothed using a Gaussian filter 4 mm full width at half maximum. All smoothed images were filtered using a typical temporal bandpass (0.01 Hz–0.1 Hz) to reduce low-frequency drift and physiological high-frequency respiratory and cardiac noise. Linear trends were removed within each time series. The covariates were regressed out from the time series of every voxel, including the white matter signal, cerebrospinal fluid signal, 24 motion parameters [28, 29], and the global signal.

### Group Independent Component Analysis

In this analysis, we used Group ICA of the fMRI Toolbox (GIFT) to pick up the resting-state network [30]. The number of components was automatically estimated by minimum description length (MDL) criteria for all participants, considering the account spatial correlation [31]. Corresponding ICA images were independently selected by visual examination and spatial correlation values between ICs and the templates from GIFT (the spatial correlation used to select the ICA components was 0.4) [30], based on the FC atlas networks of MIALAB (http://mialab.mrn.org/data/index.html).

### Brain Network Construction and Graph Theoretical Analysis

We used GRETNA, a graph theoretical network analysis toolbox for imaging the connectome, to construct the functional brain network [32]. The whole brain was divided into 90 cortical and subcortical regions according to Automated Anatomical Labeling [33], and the mean time series for each of the 90 regions was extracted. Pearson’s correlation coefficients for each pair of regions were calculated for the mean time series of all of the 90 regions, and Fisher’s Z transformation was used to turn the data into z-values which were considered to have a normal distribution. Then, according to a range of selected threshold of the relation matrix, a positive binary undirected connection functional network was constructed. However, there is no defined standard for threshold selection in the construction of a binary connection brain functional network. Therefore, sparsity was used as a range of correlation coefficient thresholds for correlation metrics, which was defined as the existing number of edges in a graph divided by the maximum possible number of edges. In accord with previous studies [17, 34], we set both the sparsity and Pearson correlation threshold of the functional network to range from 0.05 to 0.5 (in 0.05 steps), resulting in a more efficient functional network than a random network with the number of artificial edges minimized.

Graph theoretical analysis was applied to assess the topological and organizational properties within the whole brain. Topological measures can be classified into global and local/nodal measures. In this study, small-world network parameters were used to assess global measures; these included the clustering coefficient (*C*_p_), the characteristic path length (*L*_p_), the normalized clustering coefficient (γ), the normalized characteristic path length (λ), and small-worldness (σ) [19, 35]. A small-world network is characterized by a much higher *C*_p_ and a similar *L*_p_ compared with 100 matched random networks [36]. Moreover, the small-worldness, σ = γ/λ, is specially >1 for small-world networks [35]. We evaluated the centrality and clustering properties, including between centrality (BC), degree centrality (DC), nodal clustering coefficient (NCC), and nodal efficiency (NE) in local topological measures.

### Statistical Analysis

The demographic data differences between narcolepsy patients and healthy controls were computed by the independent two sample *t*-test or the χ^2^ test with the IBM Statistical Package for the Social Sciences 23.0 software (IBM SPSS Inc., Chicago, IL). We set the significance level at *P* < 0.05. Values are expressed as the mean ± SD or median (25% quartile, 75% quartile).

To test for differences in ICA networks, the one-sample *t*-test was used to obtain individual-level network homogenous maps. For each selected component, a grey mask was used to confine the one-sample test analysis to grey matter. Significant voxels (false discovery rate (FDR) corrected, *P* < 0.01) of the one-sample *t*-test results from each group were used to acquire a union mask in the between-group two-sample *t*-test. Then the two-sample *t*-test was used to test for differences in the ICA network between groups at *P* < 0.05 (FDR corrected); minimal cluster size was set to 10 voxels, with age, gender, and body mass index (BMI) as nuisance covariates.

To compare graph theoretical network properties between patients and controls, integration of the topological metric over all selected ranges of sparsity values was calculated in patients and controls using GRETNA. The two-sample *t*-test between patients and controls was performed using global or local topological measures and corrected for FDR (*P* < 0.05), with age, gender, and BMI as nuisance covariates.

In the analysis of nodal FC, the nodes showing significant differences and the same alterations in topological properties between narcolepsy patients and healthy controls were selected as ROI seeds, then FC between each pair of ROI seeds was calculated by GRETNA. The two-sample *t*-test was performed between groups and corrected by network-based statistic (*P* < 0.05), with age, gender, and body mass index (BMI) as nuisance covariates.

To determine whether the topological properties and nodal FC were correlated with the severity of sleepiness in the narcolepsy patients, a partial correlation analysis was performed to evaluate the relationship between sleepiness severity (ESS, mean sleep latency, and mean REM sleep latency from the MSLT) and topological properties in brain regions or nodal functional connectivity between ROI seeds showing significant differences between groups, adjusted for gender, age, and BMI. Partial correlation analysis was performed using the IBM Statistical Package for the Social Sciences 23.0 software (IBM SPSS Inc.) with a significance level at *P* < 0.05.

## Results

### Demographic Data

There were no significant differences between narcolepsy patients and healthy controls in age, gender, and BMI. Significant differences were found in ESS between patients and controls (Table 1).

### Independent Component Analysis and Between-Group Comparison

**Fig. 1:**
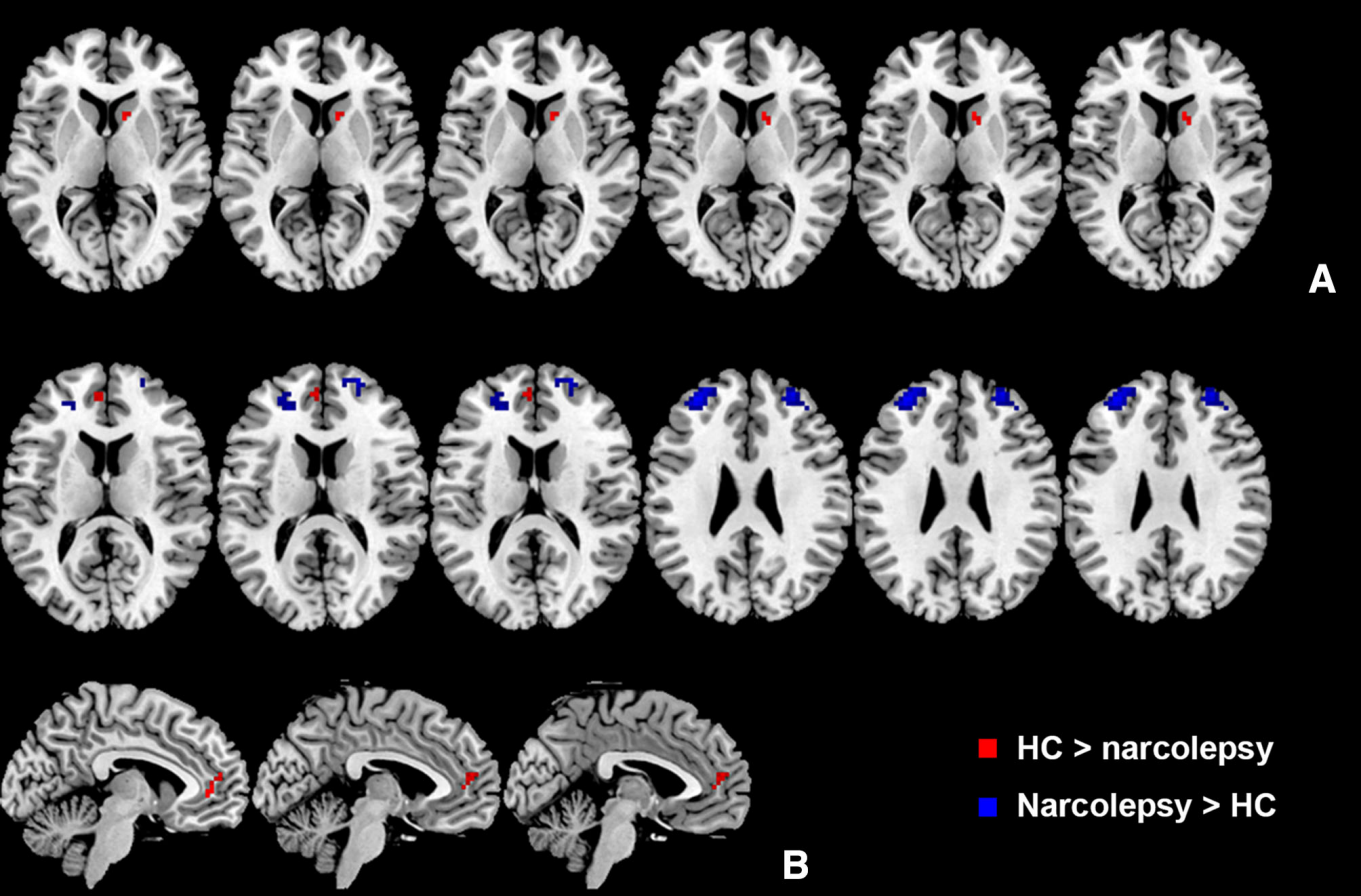
Group differences in resting-state functional connectivity from ICA between narcolepsy patients and healthy controls. **A** Salience network; **B** Executive network.

Corresponding ICA images were independently selected from the two groups according to the templates presented by GIFT. Forty-eight components were obtained, 21 of which were selected as the resting-state network: 2 components in the language network, 3 in the default mode network (DMN), 3 in the visual network, 3 in the executive network, 3 in the sensorimotor network, 2 in the salience network, 4 in the attention network, and the remaining component in the cerebellum (Fig. S1). Age, gender, and BMI were used as covariates for group comparison between patients and controls and the differences are shown in Table 2 and Fig. 1. Compared with controls, decreased FC in the right caudate within the salience network (Fig. 1A) and decreased FC in the left medial frontal gyrus within executive network (Fig. 1B) were found in narcolepsy patients, while increased FC in the bilateral middle frontal gyrus within the executive network (Fig. 1B) was also found in patients.

**Table 2:** Functional connectivity in healthy controls and narcolepsy patients from ICA results in the executive network (**A**) and the salience network (**B**).

Brain region	X (mm)	Y (mm)	Z (mm)	Size (voxels)	T value
A					
Middle frontal gyrus L	−33	48	27	56	−6.28
Middle frontal gyrus R	30	51	27	47	−5.65
Medial frontal gyrus L	−6	45	3	10	4.82
B					
Caudate nucleus R	12	15	9	10	5.45

Significant differences between healthy controls and narcolepsy patients were corrected for false discovery rate at *P* < 0.05. A positive *T* value means higher FC in controls compared with patients and a negative *T* value means higher FC in patients than in controls.

### Graph Theoretical Analysis

**Fig. 2:**
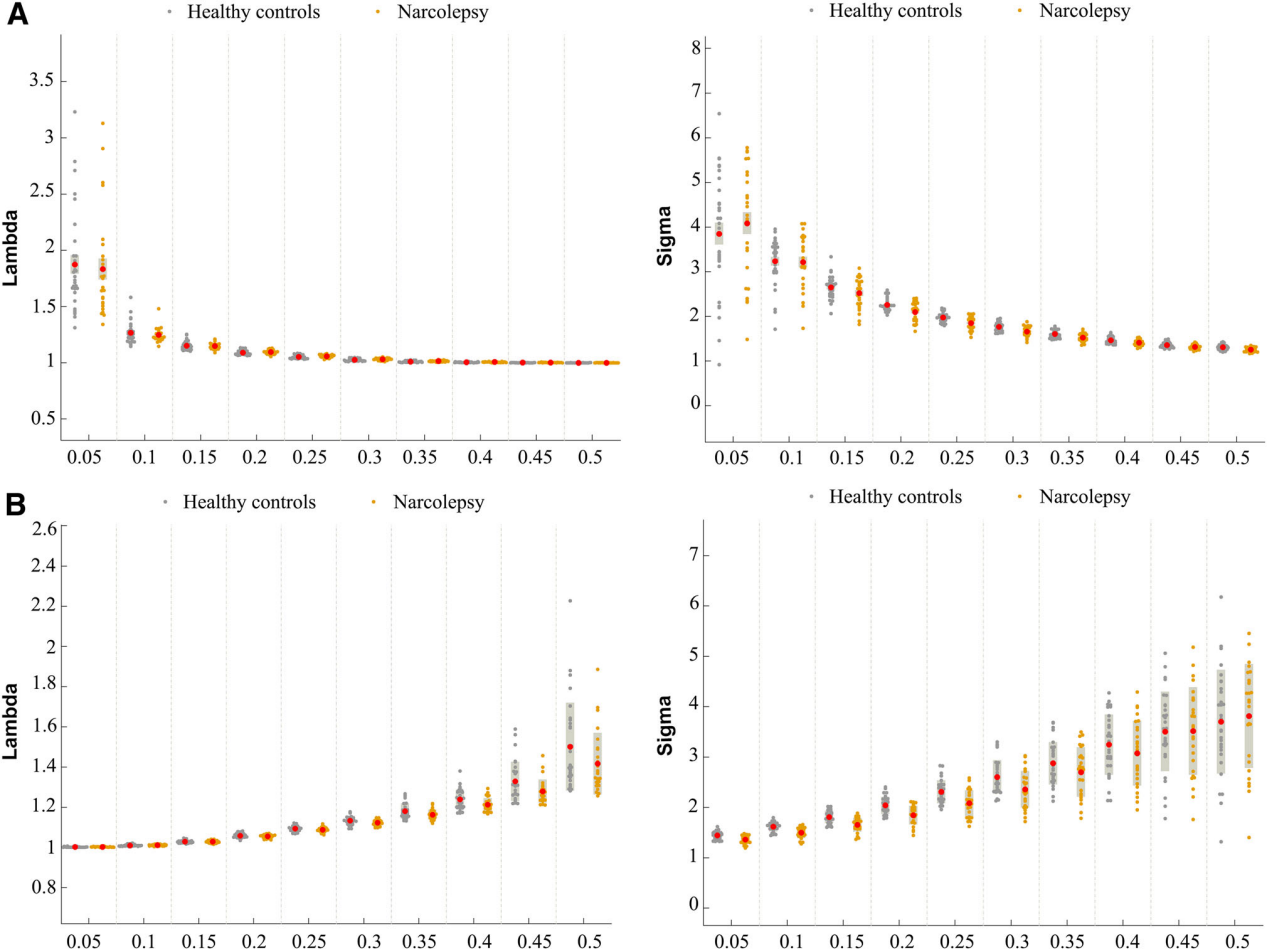
Small-world network parameters Lambda and Sigma in narcolepsy patients and healthy controls for all selected sparsity values (**A**) and Pearson correlation thresholds (**B**).

We performed graph theoretical analysis in a range of selected sparsity values or Pearson correlation thresholds in which the property of a network was considered to be credible in the result. Both the adult narcolepsy patients and healthy controls exhibited small-world topological networks (λ ≈ 1 and σ > 1) among selected sparsity values or Pearson correlation thresholds (Fig. 2). But there was no significant difference in the small-world network parameters between patients and controls among the selected sparsity values or Pearson correlation thresholds (FDR, *P* < 0.05).

**Fig. 3:**
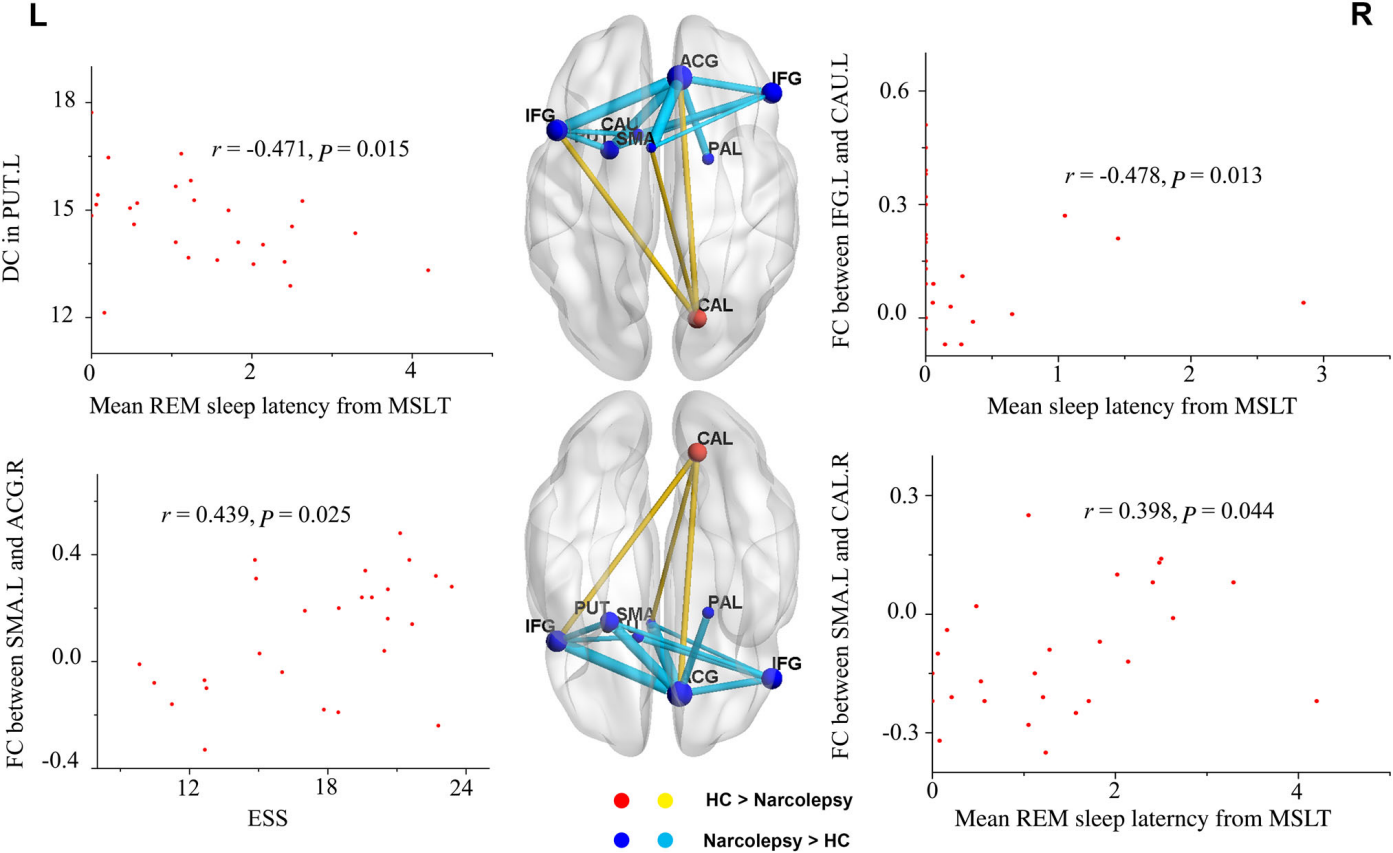
Eight overlapping regions in centrality measurements (BC and DC) and their functional connectivity in comparisons between healthy controls and narcolepsy patients. Scatter plots and partial correlation analysis between nodal topological or functional connectivity and severity of sleepiness in narcolepsy. FC, functional connectivity; ESS, Epworth Sleepiness Score; IFG, Inferior frontal gyrus; ACG, anterior cingulate gyrus; CAU, caudate nucleus; PUT, putamen; PAL, pallidum; SMA, supplementary motor area; CAL, calcarine fissure; L, left; R, right.

**Fig. 4:**
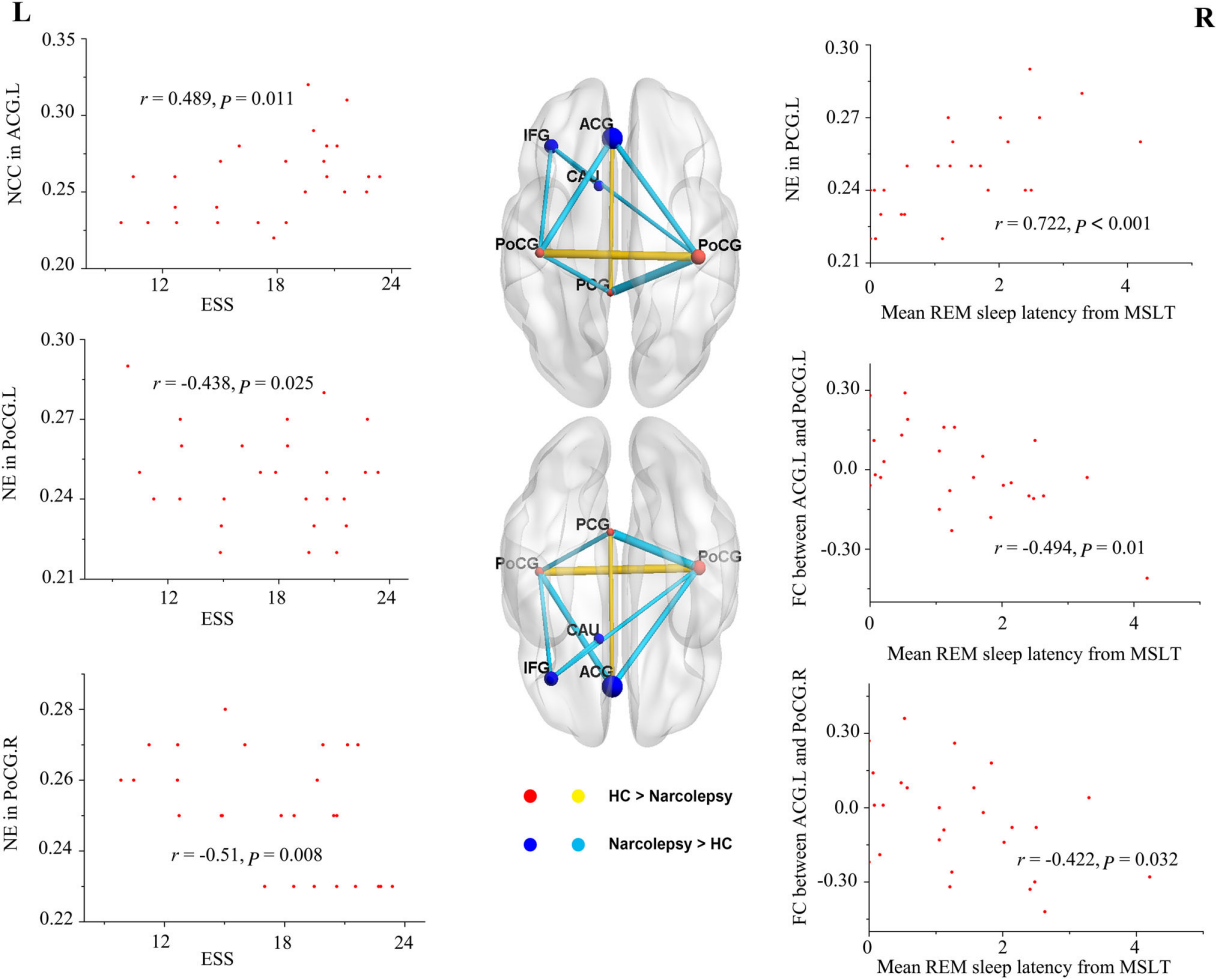
Six overlapping regions in clustering measurements (NCC and NE) and their functional connectivity in comparisons between healthy controls and narcolepsy patients. Scatter plots and partial correlation analysis between nodal topological or functional connectivity and severity of sleepiness in narcolepsy. FC, functional connectivity; ESS, Epworth Sleepiness Score; IFG, inferior frontal gyrus; ACG, anterior cingulate gyrus; CAU, caudate nucleus; PCG, posterior cingulate gyrus; PoCG, postcentral gyrus; L, left; R, right.

Sparsity values were selected in the analysis of nodal topological properties. Many brain nodes showed altered topological properties between narcolepsy patients and healthy controls. Specifically, eight overlapping nodes – the bilateral inferior frontal gyrus (IFG), right anterior cingulate gyrus (ACG), left supplementary motor area (SMA), right calcarine fissure (CAL), and bilateral basal ganglia – had the same alterations in centrality measurements (BC and DC) in patients and controls (Fig. 3 and Table 3). Meanwhile, six overlapping nodes – the left IFG, left ACG, left posterior cingulate gyrus (PCG), bilateral posterior central gyrus (PoCG), and left caudate (CAU) – had the same alterations in clustering measurements (NCC and NE) in patients and controls (Fig. 4 and Table 3).

**Table 3:** *T* values for altered overlapping areas in comparisons of the nodal topological properties between healthy controls and narcolepsy patients. **A** Altered overlapping nodes in BC and DC. **B** Altered overlapping nodes in NCC and NE.

Brain region	Side	BC	DC
A			
Inferior frontal gyrus, opercular part	L	−3.29	−4.95
Inferior frontal gyrus, triangular part	R	−2.7	−4.87
Anterior cingulate gyrus	R	−2.54	−5.87
Supplementary motor area	L	−2.5	−2.33
Calcarine fissure	R	2.83	4.46
Caudate nucleus	L	−2.38	−2.75
Putamen	L	−3.06	−4.36
Pallidum	R	−3.45	−2.8

B	Side	NCC	NE
Inferior frontal gyrus, orbital part	L	−3.69	−3.7
Anterior cingulate gyrus	L	−2.07	−5.53
Post central gyrus	L	2.79	2.49
Post central gyrus	R	3.29	3.89
Posterior cingulate gyrus	L	2.85	2.03
Caudate nucleus	L	−2.09	−2.77

Significant differences between controls and patients were corrected for false discovery rate at *P* < 0.05. A positive *T* value means higher nodal topological properties in controls than in patients and a negative *T* value means higher nodal topological properties in patients than in controls. BC, between centrality; DC, degree centrality; NCC, nodal clustering coefficient; NE, nodal efficiency; L, left; R, right.

### Nodal Functional Connectivity Analysis

Among the eight overlapping nodes in centrality differences between groups, increased FC among anterior brain areas (IFG, ACG, SMA, and basal ganglia) was found in narcolepsy patients compared with healthy controls, while decreased FC between the right CAL and some anterior brain areas (left IFG, SMA, and ACG) was detected in patients compared with controls (Fig. 3 and Table S1). Among the six overlapping nodes in clustering differences between groups, decreased FC between the left ACG and left PCG as well as FC among the bilateral PoCG was found in patients compared with controls, while FC between the left IFG or left ACG and the bilateral PoCG was higher in patients than in controls. Moreover, FC between the left PCG and bilateral PoCG as well as FC between the left IFG and left CAU was also higher in patients than in controls (Fig. 4 and Table S1).

### Partial Correlation Analysis

In the graph theoretical analysis results from narcolepsy patients, partial correlation showed that the mean REM sleep latency from MSLT was negatively correlated with DC in the left putamen (*r* = –0.471, *P* = 0.015, Fig. 3), the mean REM latency from MSLT was positively correlated with NE in the left PCG (*r* = 0.722, *P* < 0.001, Fig. 4), and the ESS was positively correlated with NCC in the left ACG (*r* = 0.489, *P* = 0.011, Fig. 4), and was negatively correlated with NE in the left PoCG (*r* = –0.438, *P* = 0.025, Fig. 4) and NE in the right PoCG (*r* = –0.51, *P* = 0.008, Fig. 4). In the FC analysis results from narcolepsy patients, the mean sleep latency was negatively correlated with FC between the left IFG and left CAU (*r* = –0.478, *P* = 0.013, Fig. 3). The mean REM sleep latency was positively correlated with FC between the left SMA and right CAL (*r* = 0.398, *P* = 0.044, Fig. 3) and the mean REM sleep latency was negatively correlated with FC between the left ACG and left PoCG (*r* = −0.494, *P* = 0.01, Fig. 4) as well as between the left ACG and right PoCG (*r* = –0.422, *P* = 0.032, Fig. 4). ESS was positively correlated with FC between the left SMA and right ACG (*r* = 0.439, *P* = 0.025, Fig. 3). Correlation analysis between nodal topological connectivity or FC and ESS among narcolepsy patients is also shown in Table S2.

## Discussion

Here, we evaluated resting FC and graph networks in adult narcolepsy patients using group ICA and graph theoretical methods. We also found that alterations in topological properties and FC within some nodes were correlated with sleepiness severity among the patients. Different from previous studies on narcolepsy in task BOLD MRI, our results demonstrated that narcolepsy patients also exhibited abnormal connectivity during resting wakefulness and some of the altered connectivity was associated with the severity of sleepiness.

### Independent Component Analysis in Comparison of Narcolepsy Patients with Healthy Controls

We found decreased FC in the left medial frontal gyrus within the executive network and decreased FC in the right caudate within the salience network in adult narcolepsy patients compared with healthy controls. Compared with controls, increased FC in the bilateral middle frontal gyrus within the executive network was detected in the narcolepsy patients.

Human narcolepsy is characterized by decreased numbers of hypocretin neurons with widespread projections throughout the whole brain, including areas involved in the executive network [37, 38]. Neuropsychological examination and brain imaging studies have indicated that narcolepsy influences executive function and emotional and cognitive processing [39–43]. Executive function requires the control and coordination of several cognitive domains including working memory and reward sensitivity [44, 45]. Both working memory and reward sensitivity are functionally coordinated by prefrontal and mesial-frontal regions [1], which are abundantly linked to the hypocretin system [2, 46]. So we infer that decreased FC in the left medial frontal gyrus within the executive network among narcolepsy patients in the present study may be associated with a reduction in hypocretin innervation within this area. Moreover, it has been reported that sleepiness due to sleep deprivation changed the FC in the medial frontal lobe [47]. Hypo-perfusion and hypo-metabolism in the medial frontal lobe have been detected in narcolepsy patients [7, 9], and they are associated with the emotional dysfunction and attention deficiency in narcolepsy. It has been shown that alteration in brain activity is not only related to disease pathology, but also depends on the conditions during imaging examination [6]. A task fMRI study showed increased brain activity in the bilateral prefrontal lobe, cingulate gyrus, thalamus, and cerebellum among adults with obstructive sleep apnea [48]. In our study, the narcolepsy patients were required to maintain wakefulness and alertness during the MRI scan, supervised by video, and this partially explains the increased FC in the bilateral middle frontal gyrus within the executive network. The increased FC in the bilateral middle frontal gyrus is in agreement with previous fMRI results in narcolepsy patients in a sustained attention condition [49].

The salience network is also involved in the maintenance of alertness and wakefulness [50, 51]. A previous sleep-deprivation study [52] showed that nocturnal sleep deprivation results in increased FC in the caudate within the salience network, which has also been found in neuroimaging studies of obstructive sleep apnea and Kleine-Levin syndrome [48, 53]. Enhanced activity in the caudate indicates monoamine release [7], which is considered to maintain alertness and wakefulness in the patients. The decreased FC in the right caudate within the salience network from our results was different from these previous studies, and the most credible explanation for this is that decreased FC in the right caudate suggests a dysfunction in wake-promotion in adult narcolepsy, which has been demonstrated in a pharmacological study of canine narcolepsy [54].

### Graph Theoretical Analysis in Comparison of Narcolepsy Patients with Healthy Controls

The adult narcolepsy patients exhibited high efficiency of small-world network properties, and there were no differences in small-world network parameters between patients and controls.

Alterations of centrality demonstrated overlapping areas in the bilateral IFG, right ACG, left SMA, bilateral basal ganglia, and right CAL between patients and controls. Excitatory hypocretin innervation of visual neurons has been described in a previous study [55], so we infer that the decreased BC and DC in the right CAL may be related to the reduced excitation by hypocretin in narcolepsy. Increased BC and DC in the right ACG is consistent with a positron emission tomography study, in which hyper-metabolism in the anterior cingulate cortex was revealed in narcolepsy patients under fully awake conditions [6]. The increased BC and DC in the bilateral IFG is partially in agreement with the ICA results from the present study, in which increased FC in the bilateral middle frontal gyrus also reflected the subjective effort to maintain alertness, and methodological differences in ICA and graph theoretical analysis led to the diversity of results in ICA and centrality measurements in the bilateral frontal lobe. Partial correlation analysis showed that DC in the left putamen was negatively correlated with mean REM sleep latency among narcolepsy patients, and this indicated that increased DC in the left putamen might be associated with the pathophysiology of sleepiness in narcolepsy.

Hypo-excitability in the sensorimotor cortex due to deficient hypocretin excitatory innervation has been reported in narcolepsy by transcranial magnetic stimulation [56], which could explain the reduced NCC and NE in the bilateral PoCG in the present study. Partial correlation analysis also suggested that NE in the bilateral PoCG was negatively correlated with ESS among patients, indicating that decreased NE in the postcentral gyrus on both sides was associated with the severity of subjective sleepiness in narcolepsy. PCG is a core node in the DMN, and a disrupted DMN has been shown to be disease-specific for narcolepsy in an EEG-fMRI study [57]. Moreover, partial correlation analysis showed that NE in the PCG is positively correlated with the mean REM sleep latency, indicating that reduced NE in the PCG was also associated with the pathophysiology of sleepiness in narcolepsy. Enhanced NE and NCC in the left ACG, left IFG, and left basal ganglia was consistent with the increased BC and DC within these areas in the present study; also, the increased NE in left ACG was correlated with the severity of subjective ESS.

### Nodal Functional Connectivity in Comparison of Narcolepsy Patients with Healthy Controls

An increased FC between the right ACG and bilateral basal ganglia (left putamen and right pallidum) was found in narcolepsy patients compared with healthy controls. Both the ACG and basal ganglia are core components of the salience network [50, 51], and increased FC among these areas is consistent with the results of previous narcolepsy studies of the salience network [48, 53]. Increased FC between the bilateral IFG and right ACG as well as the basal ganglia implies an enhanced connection between the executive and salience networks, and this might partially explain the increased FC in the bilateral frontal lobe within the executive network in our ICA results. Both increased FC within the salience network and between the executive and salience networks indicate the subjective effort to maintain wakefulness during the MRI scan [49, 52]. Especially, FC between the left IFG and left CAU was negatively correlated with REM sleep latency in narcolepsy patients, demonstrating that increased FC between the left IFG and left CAU might be associated with the pathophysiology of sleepiness in narcolepsy. The SMA is involved in motion adjustment and coordination [58]. FC between the left SMA and right ACG was positively correlated with REM sleep latency, and the FC between the left SMA and right CAL was positively correlated with ESS in narcolepsy patients, indicating that abnormal activity in the SMA and its connections might also be associated with the abnormal sleepiness in narcolepsy. Also, decreased FC between the right CAL and left IFG as well as decreased FC between the right CAL and right ACG was found in patients compared with controls. This decreased FC might be correlated with the decreased centrality in the right CAL just as in the graph theoretical analysis results in the present study.

Decreased FC among the bilateral PoCG was in accord with the changes in topological properties in the bilateral PoCG, which is associated with hypo-excitability within these areas due to hypocretin dysfunction [56]. Decreased FC between the left PCG and left ACG demonstrated a potential dysfunctional connection between the salience network and the DMN; this has rarely been reported in previous studies. FC between the left ACG and bilateral PoCG was negatively correlated with REM sleep latency, which implied that increased FC between the left ACG and bilateral PoCG might also be associated with the pathophysiology of sleepiness in narcolepsy. Increased FC between the left PCG and bilateral PoCG might be a compensation for decreased NE/NCC within these areas in narcolepsy patients.

Increased ESS, and shortened sleep latency and REM sleep latency are all parameters that indicate the severity of narcolepsy. Also, a shortened REM sleep latency is a clinical characteristic of narcolepsy. In our results, for nodal topological properties, the NCC value in the left ACG and the NE value in the bilateral PoCG were each correlated with ESS, suggesting that subjective sleepiness measurement is strongly associated with network alterations in these brain areas. The DC value in the left PUT and the NE in the left PCG were each correlated with REM sleep latency, suggesting that network changes in both areas may be a specific indicator for evaluating the severity of narcolepsy. As for FC, an increase between the left SMA and right ACG was positively correlated with ESS, implying subjective sleepiness measurement is also associated with altered nodal connectivity. Increased FC between the left IFG and left CAU was negatively correlated with sleep latency, indicating that the FC changes between the frontal cortex and basal ganglia may be one of the parameters describing the severity of narcolepsy. Meanwhile, the decreased FC between the left SMA and right CAL was positively correlated with REM sleep latency, and the increased FC between the left ACG and bilateral PoCG was negatively correlated with REM sleep latency, indicating that altered FC may also be a specific indicator for evaluating the severity of narcolepsy.

Limitations of this study should be considered. Although the maintenance of wakefulness during the MRI examination was controlled clinically and supervised by video, the alertness of narcolepsy patients should be monitored by simultaneous EEG during the fMRI. In previous neuroimaging studies of narcolepsy, the maintenance of wakefulness during a scan was also resolved by clinical supervision [6–9]. Considering the diagnostic specificity in cerebrospinal fluid hypocretin measurements for narcolepsy, the absence of hypocretin measurements is also a limitation of the present study. Moreover, it is essential to compare narcolepsy with other somnolence disorders and sleep deprivation in the future.

## Conclusions

In this resting-state ICA and graph theoretical study in adult narcolepsy patients, we found altered connectivity within the executive and salience networks by ICA. Functional connection changes between the left frontal cortex and left basal ganglia may be one of the parameters describing the severity of narcolepsy. Alterations in nodal topological properties in the left putamen and left posterior cingulate, changes in FC between the left supplementary motor area and right occipital as well as changes in FC between the left anterior cingulate gyrus and bilateral postcentral gyrus can be considered specific indicators for evaluating the severity of narcolepsy.

## Electronic supplementary material

Below is the link to the electronic supplementary material.

Supplementary material 1 (PDF 111 kb)

## References

[1] Moraes M, Rossini S, Reimao R (2012). Executive attention and working memory in narcoleptic outpatients. Arq Neuropsiquiatr.

[2] Zhang XY, Yu L, Zhuang QX, Zhu JN, Wang JJ (2013). Central functions of the orexinergic system. Neurosci Bull.

[3] Nishino S, Okuro M, Kotorii N, Anegawa E, Ishimaru Y, Matsumura M (2010). Hypocretin/orexin and narcolepsy: new basic and clinical insights. Acta Physiol (Oxf).

[4] Peyron C, Faraco J, Rogers W, Ripley B, Overeem S, Charnay Y (2000). A mutation in a case of early onset narcolepsy and a generalized absence of hypocretin peptides in human narcoleptic brains. Nat Med.

[5] Thannickal TC, Moore RY, Nienhuis R, Ramanathan L, Gulyani S, Aldrich M (2000). Reduced number of hypocretin neurons in human narcolepsy. Neuron.

[6] Dauvilliers Y, Comte F, Bayard S, Carlander B, Zanca M, Touchon J (2010). A brain PET study in patients with narcolepsy-cataplexy. J Neurol Neurosurg Psychiatry.

[7] Joo EY, Hong SB, Tae WS, Kim JH, Han SJ, Cho YW (2005). Cerebral perfusion abnormality in narcolepsy with cataplexy. Neuroimage.

[8] Huang YS, Liu FY, Lin CY, Hsiao IT, Guilleminault C (2016). Brain imaging and cognition in young narcoleptic patients. Sleep Med.

[9] Joo EY, Tae WS, Kim JH, Kim BT, Hong SB (2004). Glucose hypometabolism of hypothalamus and thalamus in narcolepsy. Ann Neurol.

[10] Desseilles M, Dang-Vu T, Schabus M, Sterpenich V, Maquet P, Schwartz S (2008). Neuroimaging insights into the pathophysiology of sleep disorders. Sleep.

[11] Wang W, Li Q, Wang Y, Tian J, Yang W, Li W (2011). Brain fMRI and craving response to heroin-related cues in patients on methadone maintenance treatment. Am J Drug Alcohol Abuse.

[12] Fox MD, Raichle ME (2007). Spontaneous fluctuations in brain activity observed with functional magnetic resonance imaging. Nat Rev Neurosci.

[13] Beckmann CF, DeLuca M, Devlin JT, Smith SM (2005). Investigations into resting-state connectivity using independent component analysis. Philos Trans R Soc Lond B Biol Sci.

[14] McKeown MJ, Sejnowski TJ (1998). Independent component analysis of fMRI data: examining the assumptions. Hum Brain Mapp.

[15] Calhoun VD, Liu J, Adali T (2009). A review of group ICA for fMRI data and ICA for joint inference of imaging, genetic, and ERP data. Neuroimage.

[16] Bassett DS, Bullmore ET (2009). Human brain networks in health and disease. Curr Opin Neurol.

[17] Zhao X, Tian L, Yan J, Yue W, Yan H, Zhang D (2017). Abnormal rich-club organization associated with compromised cognitive function in patients with schizophrenia and their unaffected parents. Neurosci Bull.

[18] Dennis EL, Thompson PM (2014). Functional brain connectivity using fMRI in aging and Alzheimer’s disease. Neuropsychol Rev.

[19] Rubinov M, Sporns O (2010). Complex network measures of brain connectivity: uses and interpretations. Neuroimage.

[20] Engstrom M, Hallbook T, Szakacs A, Karlsson T, Landtblom AM (2014). Functional magnetic resonance imaging in narcolepsy and the kleine-levin syndrome. Front Neurol.

[21] Wada M, Mimura M, Noda Y, Takasu S, Plitman E, Honda M (2018). Neuroimaging correlates of narcolepsy with cataplexy: A systematic review. Neurosci Res.

[22] Medicine AAoS. The International Classification of Sleep Disorders. 3rd edn. Westchester, 2014.

[23] Billings ME, Rosen CL, Auckley D, Benca R, Foldvary-Schaefer N, Iber C (2014). Psychometric performance and responsiveness of the functional outcomes of sleep questionnaire and sleep apnea quality of life instrument in a randomized trial: the HomePAP study. Sleep.

[24] Berry RB, Budhiraja R, Gottlieb DJ, Gozal D, Iber C, Kapur VK*, et al.* Rules for scoring respiratory events in sleep: update of the 2007 AASM Manual for the Scoring of Sleep and Associated Events. Deliberations of the Sleep Apnea Definitions Task Force of the American Academy of Sleep Medicine. J Clin Sleep Med 2012, 8: 597–619. 10.5664/jcsm.2172 PMC3459210 23066376

[25] Littner MR, Kushida C, Wise M, Davila DG, Morgenthaler T, Lee-Chiong T (2005). Practice parameters for clinical use of the multiple sleep latency test and the maintenance of wakefulness test. Sleep.

[26] Dauvilliers Y, Evangelista E, de Verbizier D, Barateau L, Peigneux P (2017). [18F]Fludeoxyglucose-positron emission tomography evidence for cerebral hypermetabolism in the awake state in narcolepsy and idiopathic hypersomnia. Front Neurol.

[27] Yan CG, Wang XD, Zuo XN, Zang YF (2016). DPABI: data processing & analysis for (resting-state) brain imaging. Neuroinformatics.

[28] Friston KJ, Williams S, Howard R, Frackowiak RS, Turner R (1996). Movement-related effects in fMRI time-series. Magn Reson Med.

[29] Yan CG, Craddock RC, He Y, Milham MP (2013). Addressing head motion dependencies for small-world topologies in functional connectomics. Front Hum Neurosci.

[30] Calhoun VD, Adali T, Pearlson GD, Pekar JJ (2001). A method for making group inferences from functional MRI data using independent component analysis. Hum Brain Mapp.

[31] Li W, Li Q, Wang D, Xiao W, Liu K, Shi L (2015). Dysfunctional default mode network in methadone treated patients who have a higher heroin relapse risk. Sci Rep.

[32] Wang J, Wang X, Xia M, Liao X, Evans A, He Y (2015). Corrigendum: GRETNA: a graph theoretical network analysis toolbox for imaging connectomics. Front Hum Neurosci.

[33] Tzourio-Mazoyer N, Landeau B, Papathanassiou D, Crivello F, Etard O, Delcroix N (2002). Automated anatomical labeling of activations in SPM using a macroscopic anatomical parcellation of the MNI MRI single-subject brain. Neuroimage.

[34] Achard S, Bullmore E (2007). Efficiency and cost of economical brain functional networks. PLoS Comput Biol.

[35] Watts DJ, Strogatz SH (1998). Collective dynamics of ‘small-world’ networks. Nature.

[36] Zhong Z, Zhao T, Luo J, Guo Z, Guo M, Li P (2014). Abnormal topological organization in white matter structural networks revealed by diffusion tensor tractography in unmedicated patients with obsessive-compulsive disorder. Prog Neuropsychopharmacol Biol Psychiatry.

[37] Collette F, Van der Linden M (2002). Brain imaging of the central executive component of working memory. Neurosci Biobehav Rev.

[38] Collette F, Van der Linden M, Laureys S, Delfiore G, Degueldre C, Luxen A (2005). Exploring the unity and diversity of the neural substrates of executive functioning. Hum Brain Mapp.

[39] Naumann A, Bellebaum C, Daum I (2006). Cognitive deficits in narcolepsy. J Sleep Res.

[40] Ponz A, Khatami R, Poryazova R, Werth E, Boesiger P, Bassetti CL (2010). Abnormal activity in reward brain circuits in human narcolepsy with cataplexy. Ann Neurol.

[41] Ponz A, Khatami R, Poryazova R, Werth E, Boesiger P, Schwartz S (2010). Reduced amygdala activity during aversive conditioning in human narcolepsy. Ann Neurol.

[42] Rieger M, Mayer G, Gauggel S (2003). Attention deficits in patients with narcolepsy. Sleep.

[43] Schwartz S, Ponz A, Poryazova R, Werth E, Boesiger P, Khatami R (2008). Abnormal activity in hypothalamus and amygdala during humour processing in human narcolepsy with cataplexy. Brain.

[44] Delazer M, Hogl B, Zamarian L, Wenter J, Gschliesser V, Ehrmann L (2011). Executive functions, information sampling, and decision making in narcolepsy with cataplexy. Neuropsychology.

[45] Wu W, Cui L, Fu Y, Tian Q, Liu L, Zhang X (2016). Sleep and cognitive abnormalities in acute minor thalamic infarction. Neurosci Bull.

[46] Korotkova TM, Sergeeva OA, Eriksson KS, Haas HL, Brown RE (2003). Excitation of ventral tegmental area dopaminergic and nondopaminergic neurons by orexins/hypocretins. J Neurosci.

[47] Shao Y, Wang L, Ye E, Jin X, Ni W, Yang Y (2013). Decreased thalamocortical functional connectivity after 36 hours of total sleep deprivation: evidence from resting state FMRI. PLoS One.

[48] Ayalon L, Ancoli-Israel S, Klemfuss Z, Shalauta MD, Drummond SP (2006). Increased brain activation during verbal learning in obstructive sleep apnea. Neuroimage.

[49] Thomas RJ (2005). Fatigue in the executive cortical network demonstrated in narcoleptics using functional magnetic resonance imaging–a preliminary study. Sleep Med.

[50] Uddin LQ (2015). Salience processing and insular cortical function and dysfunction. Nat Rev Neurosci.

[51] Spetsieris PG, Ko JH, Tang CC, Nazem A, Sako W, Peng S (2015). Metabolic resting-state brain networks in health and disease. Proc Natl Acad Sci U S A.

[52] Fang Z, Spaeth AM, Ma N, Zhu S, Hu S, Goel N (2015). Altered salience network connectivity predicts macronutrient intake after sleep deprivation. Sci Rep.

[53] Dauvilliers Y, Bayard S, Lopez R, Comte F, Zanca M, Peigneux P (2014). Widespread hypermetabolism in symptomatic and asymptomatic episodes in Kleine-Levin syndrome. PLoS One.

[54] Kanbayashi T, Honda K, Kodama T, Mignot E, Nishino S (2000). Implication of dopaminergic mechanisms in the wake-promoting effects of amphetamine: a study of D- and L-derivatives in canine narcolepsy. Neuroscience.

[55] Bayer L, Serafin M, Eggermann E, Saint-Mleux B, Machard D, Jones BE (2004). Exclusive postsynaptic action of hypocretin-orexin on sublayer 6b cortical neurons. J Neurosci.

[56] Oliviero A, Della Marca G, Tonali PA, Pilato F, Saturno E, Dileone M (2005). Functional involvement of cerebral cortex in human narcolepsy. J Neurol.

[57] Drissi NM, Szakacs A, Witt ST, Wretman A, Ulander M, Stahlbrandt H (2016). Altered brain microstate dynamics in adolescents with narcolepsy. Front Hum Neurosci.

[58] Diez-Cirarda M, Strafella AP, Kim J, Pena J, Ojeda N, Cabrera-Zubizarreta A (2018). Dynamic functional connectivity in Parkinson’s disease patients with mild cognitive impairment and normal cognition. Neuroimage Clin.

