# Supplementary material for: Independent Component Analysis and Graph Theoretical Analysis in Patients with Narcolepsy
Source: Neurosci Bull. 2018 Nov 13;35(4):743–55. doi: 10.1007/s12264-018-0307-6 (PMC6616568; doi:10.1007/s12264-018-0307-6)
Supplement: Supplementary file 1 — Supplementary material 1 (PDF 111 kb) [file 12264_2018_307_MOESM1_ESM.pdf]

Supplementary Material

Independent Component Analysis and Graph Theoretical Analysis in Patients with Narcolepsy

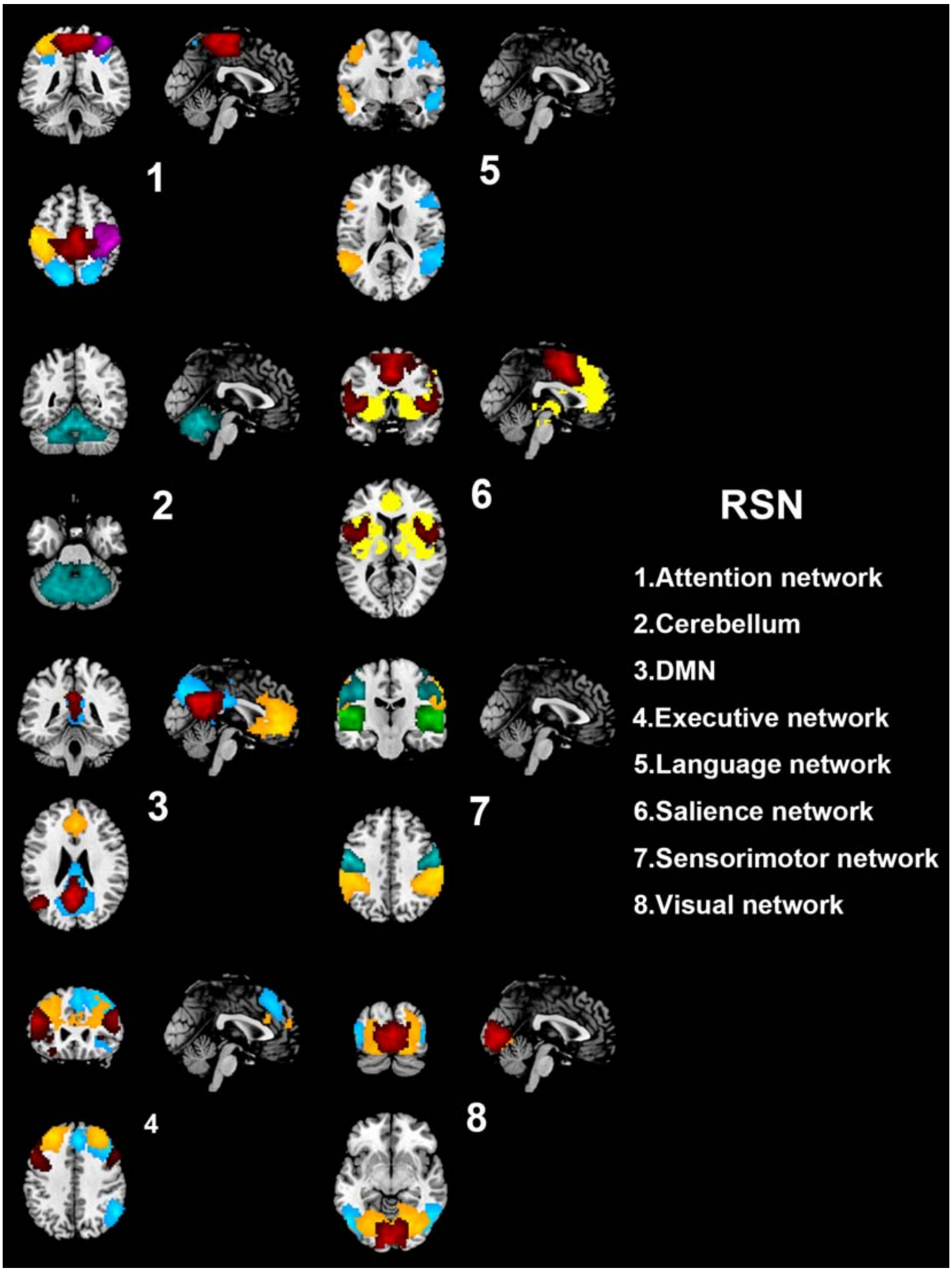

Figure S1 ICA results showing that a total of 8 resting-state networks were detected.

Table S1 T values for differences in functional connectivity between healthy controls and narcolepsy patients

Functional connectivity	T value
Between IFG.L and SMA.L	–2.84
Between IFG.L and ACG.R	–5.57
Between IFG.L and CAL.R	2.75
Between IFG.L and CAU.L	–2.34
Between IFG.L and PUT.L	–2.95
Between IFG.L and PoCG.L	–2.19
Between IFG.L and PoCG.R	–2.1
Between IFG.R and SMA.L	–2.02
Between IFG.R and ACG.R	–3.52
Between IFG.R and CAU.L	–3.47
Between IFG.R and PUT.L	–2.15
Between SMA.L and ACG.R	–5.58
Between SMA.L and CAL.R	2.22
Between ACG.L and PCG.L	2.27
Between ACG.L and PoCG.L	–3.29
Between ACG.L and PoCG.L	–3.00
Between ACG.R and CAL.R	2.85
Between ACG.R and PUT.L	–5.00
Between ACG.R and PAL.R	–3.51

Between PCG.L and PoCG.L	–2.49
Between PCG.L and PoCG.R	–4.45
Between PoCG.L and PoCG.R	4.39

Significant differences between healthy controls and narcolepsy patients was corrected for the network-based statistic at *P*<0.05. A positive T value means higher FC in controls than in patients and a negative T value means higher FC in patients than in controls. FC, functional connectivity; IFG, inferior frontal gyrus; ACG, anterior cingulate gyrus; SMA, supplementary motor area; CAL, calcarine fissure; PCG, posterior cingulate gyrus; PoCG, posterior central gyrus; CAU, caudate; PUT, putamen; PAL, pallidum.

Table S2 Correlation analysis between nodal topological or functional connectivity and severity of sleepiness among narcolepsy patients before and after adjusting for covariates

	**Non-adjusted**		**Adjusted**	
	r	***P***	***r***	***P***
Between DC in PUT.L and mean REM sleep latency#	–0.254	0.211	–0.471	0.015
Between NCC in ACG.L and ESS*	0.437	0.025	0.489	0.011
Between NE in PCG.L and mean REM sleep latency#	0.265	0.191	0.722	<0.001
Between NE in PoCG.L and ESS*	–0.283	0.161	–0.438	0.025
Between NE in PoCG.R and ESS*	–0.373	0.061	–0.51	0.008
Between FC IFG.L-CAU.L and mean sleep latency#	–0.361	0.07	–0.478	0.013
Between FC SMA.L-ACG.R and ESS#	0.377	0.057	0.439	0.025

Between FC SMA.L-CAL.R and mean REM sleep latency#	0.195	0.34	0.398	0.044
Between FC ACG.L-PoCG.L and mean REM sleep latency#	–0.154	0.452	–0.494	0.01
Between FC ACG.L-PoCG.R and mean REM sleep latency#	–0.215	0.29	–0.422	0.032

Adjusted for age, gender, and body mass index. r, Pearson correlation coefficient (*) or Spearman correlation coefficient (#); ESS, Epworth Sleepiness Score; BC, between centrality; DC, degree centrality; NCC, nodal clustering coefficient; NE, nodal efficiency; PUT, putamen; ACG, anterior cingulate gyrus; PCG, posterior cingulate gyrus; PoCG, post central gyrus; IFG, inferior frontal gyrus; CAU, caudate; SMA, supplementary motor area; CAL, calcarine fissure; L, left; R, right; FC IFG.L-CAU.L. functional connectivity between IFG.L and CAU.L
